# Barriers and Facilitators to Timely Detection and Optimal Management of Chronic Wet Cough in Aboriginal Children Across Australia From the Perspective of Health Care Providers: A Qualitative Study

**DOI:** 10.5694/mja2.70312

**Published:** 2026-09-30

**Authors:** Gloria T. Y. Lau, Pamela Laird, Robyn Aitken, Melanie Barwick, Liam Bedford, Emily R. Bowden, Corey Dalton, Jamie Everingham, Angela Fuery, Reece Griffin, Neha Jain, Mina Kinghorn, Renae Knapp, Gabrielle B. McCallum, Peter Morris, Richard Norman, Peter C. Richmond, Aarti Saiganesh, Slade Sibosado, Maree Toombs, Roz Walker, Joan Wilson, Anne B. Chang, André Schultz

**Affiliations:** ^1^ The Kids Research Institute Perth Western Australia Australia; ^2^ Perth Children's Hospital Perth Western Australia Australia; ^3^ The University of Western Australia Perth Western Australia Australia; ^4^ Flinders University Darwin Northern Territory Australia; ^5^ Hospital for Sick Children Toronto Ontario Canada; ^6^ University of Toronto Toronto Ontario Canada; ^7^ The Kids Kimberley The Kids Research Institute Broome Western Australia Australia; ^8^ Charles Darwin University Darwin Northern Territory Australia; ^9^ Derbarl Yerrigan Health Service Perth Western Australia Australia; ^10^ Carbal Medical Services Toowoomba Queensland Australia; ^11^ University of Queensland Brisbane Queensland Australia; ^12^ Royal Darwin Hospital Darwin Northern Territory Australia; ^13^ Curtin University Perth Western Australia Australia; ^14^ Kimberley Aboriginal Medical Service Broome Western Australia Australia; ^15^ University of Sydney Sydney New South Wales Australia; ^16^ Murdoch University Perth Western Australia Australia; ^17^ Yura Yungi Medical Service Aboriginal Corporation Halls Creek Western Australia Australia; ^18^ Australian Centre for Health Services Innovation and School of Clinical Medicine Queensland University of Technology Brisbane Queensland Australia

**Keywords:** child health, childhood diseases, primary care, respiratory tract infections

## Abstract

**Objective:**

To identify barriers and facilitators to the timely detection and optimal management of chronic wet cough (CWC) in children from the perspective of health care providers (HCPs) across primary care clinics providing services to Australian Aboriginal people in multiple, diverse communities.

**Study Type:**

Qualitative participatory action research study.

**Setting and Participants:**

Eight Australian primary care clinics across three states and territories. Semi‐structured interviews and focus groups were conducted with 83 HCPs from eight primary care clinics across metropolitan, regional and remote health service settings between May 2021 and July 2023.

**Main Outcome Measures:**

Barriers to, and facilitators of, detection and optimal management of CWC in Aboriginal children in primary care settings.

**Results:**

Key barriers identified included limited condition‐specific knowledge, time constraints and competing priorities. HCPs identified key facilitators, such as the need for additional training, educational resources and various modifications to clinic systems. Barriers and facilitators were similar across settings. Proposed solutions varied and were based on several factors, such as community size, availability of existing infrastructure and local preferences. In addition, HCPs highlighted the importance of a culturally secure environment in fostering engagement and communication with Aboriginal families.

**Conclusion:**

Although the barriers and facilitators to the detection and management of CWC in Aboriginal children were largely consistent across settings, solutions must be tailored to each community's unique circumstances. A comprehensive approach is required to overcome barriers and deliver the intervention within a culturally secure framework.

## Plan Language Summary

**The known:** Chronic wet cough (CWC) is highly prevalent in Aboriginal and Torres Strait Islander children. However, there is limited clinician awareness of CWC. Optimising management of CWC improves clinical outcomes and quality of life.

**The new:** Clinicians from eight sites across three Australian states/territories identified the need for additional training, a variety of educational resources and several modifications to clinic systems to facilitate the detection and optimal management of CWC.

**The implications:** Specific solutions must be tailored to each site's unique circumstances. Ensuring a culturally secure clinical environment is essential to improve the detection and optimal management of CWC in primary care.

## 1 Introduction

Chronic wet cough (CWC) is highly prevalent among Aboriginal and Torres Strait Islander children [1] and is often caused by protracted bacterial bronchitis (PBB), which can progress to life‐limiting disease (bronchiectasis) if untreated [2]. Primary health care clinics have a crucial role in detecting CWC, which allows for timely PBB management and, in turn, can prevent the progression of PBB to bronchiectasis. Therefore, long‐term lung health trajectories can be improved through timely detection and management in primary care. Arguably, primary care clinicians caring for Aboriginal and Torres Strait Islander children should be proficient in detecting and managing CWC; however, it is often under‐recognised [3] and PBB inadequately treated [3]. The challenge is pronounced in Aboriginal and Torres Strait Islander children, where complex cultural and socio‐economic factors interact with health system factors [3, 4].

We previously studied barriers and facilitators to timely detection and management of childhood CWC in a regional town in Western Australia [3, 4], followed by implementing a program comprising knowledge dissemination for carers, clinician education and clinic practice changes [5]. The program successfully improved CWC detection and management and cough‐specific health outcomes. Given Australia's cultural and geographical diversity and high rates of chronic respiratory disease [1], there is a need to determine how barriers and facilitators for detection and management of childhood CWC might vary across different settings.

Our study aimed to identify barriers and facilitators to timely detection and optimal management of CWC in primary care settings from the perspective of health care providers (HCPs) to Australian Aboriginal and Torres Strait Islander children in multiple diverse communities.

## 2 Method

### 2.1 Study Design

We conducted a multi‐centre qualitative study with a participatory action research approach [6], characterised by an iterative, reflexive methodology that engages participants as collaborators. Although Indigenous Australians consist of both Aboriginal and Torres Strait Islander people, the communities included in this study are predominantly Aboriginal and, therefore, prefer to be described as Aboriginal. Hence, we used this term throughout this manuscript when referring to the study population.

Before commencing the research, extensive community engagement was conducted to ensure the study was aligned with local needs and priorities. The research involved semi‐structured interviews and focus groups with HCPs involved in the primary medical care of Aboriginal children, followed by thematic analysis. Concurrently, we conducted interviews with carers of Aboriginal children; these data were reported separately to ensure that carers' perspectives are represented distinctly [7].

The CONSIDER reporting criteria checklist for health research involving Indigenous peoples was completed for this article and can be found in Section S1 [8].

### 2.2 Study Setting

Eight Australian primary health sites participated across three states and territories (Table 1). Seven of the eight clinics were managed by Aboriginal Community Controlled Health Organisations (ACCHOs), whereas the clinic in Community 6 was government controlled. The Aboriginal Medical Services were located in communities representing a wide diversity of size (from ~100 people to ≥ 2 million people), geography and climate (from inland desert to coastal tropical) [9, 10, 11]. The proportion of Aboriginal population in the communities ranged from 2% to 95% [12, 13, 14].

**TABLE 1:** Geographical settings of the study sites.

Site	Region, state	Remoteness	Environment/climate [9, 10, 11]	Total population (Aboriginal population) [9, 10, 11]	Number of Aboriginal Medical Services clinics
1	Kimberley, Western Australia	Very remote	Desert/arid	472 (430; 91%)	1
2^a^	Kimberley, Western Australia	Very remote	Desert/arid	(111)	1
3	Kimberley, Western Australia	Very remote	Desert/arid	202 (178; 88%)	1
4	Kimberley, Western Australia	Remote	Semi‐arid	3603 (2806; 78%)	1
5	Perth, Western Australia	Major city	Mediterranean	2,116,647 (42,083; 2%)	4
6	Victoria Daly, Northern Territory	Remote	Tropical	411 (390; 95%)	1
7	East Arnhem, Northern Territory	Very remote	Tropical	705 (645; 91%)	1
8	Darling Downs, Queensland	Inner regional	Humid subtropical	280,000 (17,000; 6%) [10]	1

^a^ No data were available for the total population in Community 2.

### 2.3 Interview Guide Development

Interview guides from the team's previous interview guides on lung health were used (Table S1) [3, 4]. Interviews were also conducted to examine barriers and facilitators to implementation of a program to facilitate CWC detection and management; however, these findings are reported separately.

### 2.4 Selection Criteria and Recruitment

The Consolidated Framework for Implementation Research (CFIR) [15] guided identification of stakeholder groups (Table 2). Any HCPs involved in the care of Aboriginal children in primary care settings were eligible participants. HCPs were included in Groups 2–5, as outlined in Table 2. The perspectives of Group 1, carers of Aboriginal children, are reported separately [7].

**TABLE 2:** Stakeholder groups based on relevant Consolidated Framework for Implementation Research (CFIR) domains.

Inner setting (i.e., internal factors within a community): Carers of Aboriginal children Providers of primary care health care to Aboriginal children (e.g., doctors, nurses, Aboriginal Health Workers and physiotherapists) Primary care managerial staff (e.g., managers and board members)
Outer setting (i.e., external factors beyond the community): Paediatricians who provide a service to Aboriginal children Policymakers and guideline providers

Participants were recruited through purposive sampling [16] and snowball techniques [16], with verbal or email invitations, to ensure a wide range of roles and experiences were captured. The anticipated number of participants was estimated based on concept of information power [17], with ~2–3 focus groups of 6–8 participants and ~10 individual interviews per site. Recruitment continued until data saturation [16]. Interviews and focus groups were conducted between May 2021 and July 2023. Details about interview periods and interviewers are presented in Table S2.

Interviews were typically conducted in pairs by interviewers with qualitative research skills. Interviews occurred in the Aboriginal Medical Services, at the interviewee's workplace or via video call. Informed consent was obtained at the time of the interviews. Interview notes (handwritten/typed) or audio recordings were taken, with recordings later transcribed. Interview notes were reviewed for inconsistencies between interviewers and clarification was sought from interviewees when necessary. Interviews typically lasted ~5–15 min, with some up to 60 min. Field notes were taken during the interview or shortly after (due to time restrictions).

### 2.5 Data Analysis and Management

A reflexive thematic analysis, based on Braun and Clarke's six‐phase approach [18], was used to analyse the data for each site. Given the relative proximity of Sites 1–3 and the mobility of HCPs across these three communities, data for these sites were combined. At least three researchers conducted each site's data analysis (example illustrated in Figure 1). The analysis followed both an inductive and deductive approach, drawing on the research team's previous work [3, 4]. Each phase of analysis adhered to Lincoln and Guba's criteria of trustworthiness [19]. Data were managed using QSR NVivo Release 1 [20], with de‐identified data held on password‐protected institutional servers. The data were presented to key community members at each site after analysis completion, allowing for review and feedback.

**FIGURE 1:**
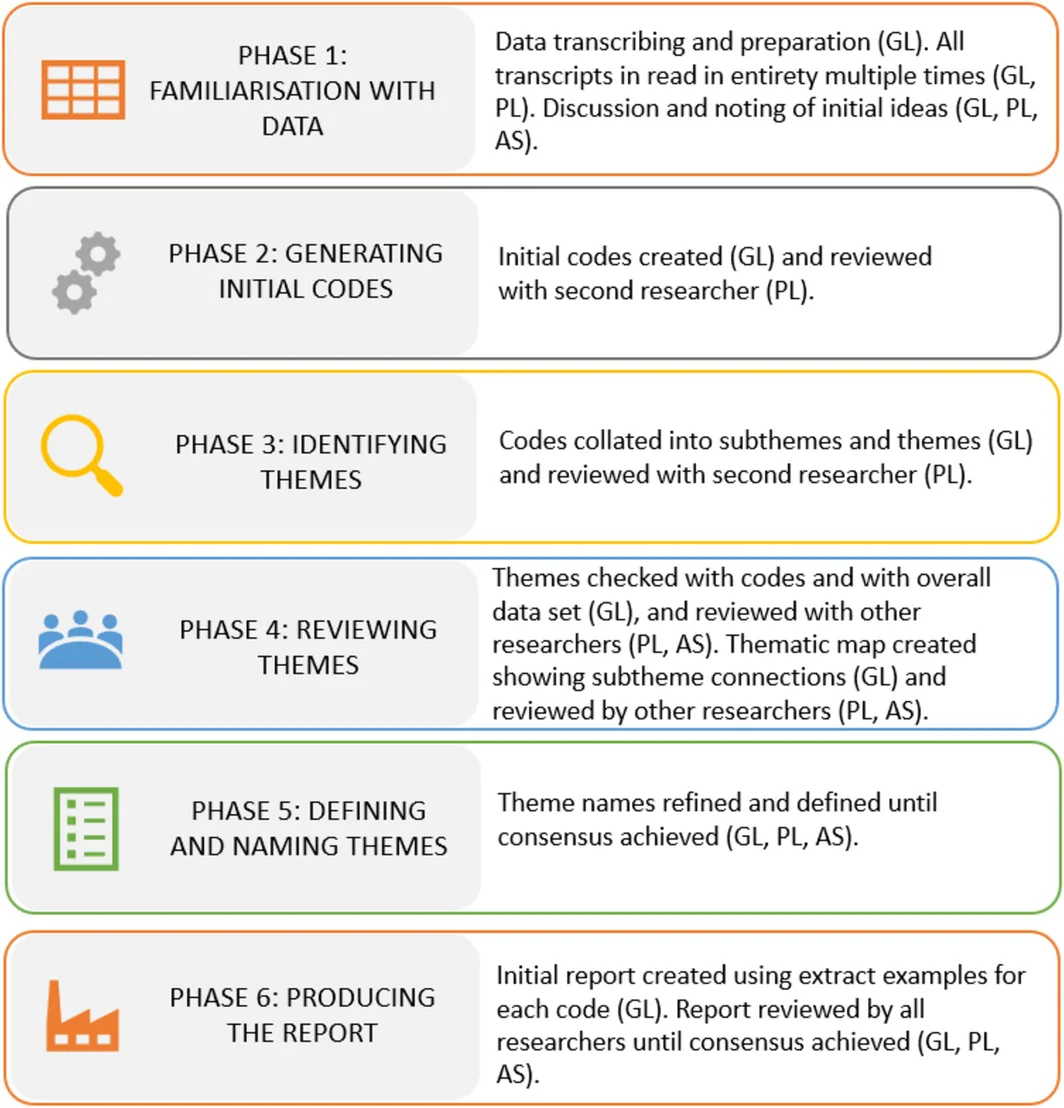
Data analysis process for the East Kimberley sites. AS, André Schultz; GL, Gloria T. Y. Lau; PL, Pamela Laird.

### 2.6 Ethics Statement

Ethics approval was obtained from Western Australian Health Child and Adolescent Health Service Ethics Committee (RGS 4136), Western Australian Aboriginal Health Ethics Committee (HREC 774), Human Research Ethics Committee of the Northern Territory Department of Health and Menzies School of Health Research (HREC 2021‐3954), Miwatj Research and Ethics Advisory Group and reciprocal ethics was given by the University of Queensland Human Research Ethics Committee (2020002563). Our study was reported according to the Consolidated Criteria for Reporting Qualitative Research (COREQ; Table S3) [21].

## 3 Results

Eighty‐three HCPs participated in interviews and/or focus groups across all sites, representing diverse roles (Table 3). Most HCPs invited agreed to participate. Those who declined indicated a lack of time or were unavailable due to clinical demands. Since HCPs were not given individual invitations, recording the precise number of individuals who declined was not practically feasible. Six focus group participants also completed individual interviews for deeper insights.

**TABLE 3:** Participant numbers.

Site	Region, state	Participant numbers	Participant roles
1	Kimberley, Western Australia	29 HCPs	General practitioners: 4 Nurses: 15 AHWs/AHPs: 2 Allied health: 1 Clinical students: 3 Managerial or executive staff: 2 Administrative staff: 2
2	Kimberley, Western Australia		
3	Kimberley, Western Australia		
4	Kimberley, Western Australia	10 HCPs	General practitioners: 1 Nurses: 2 AHWs/AHPs: 2 Aboriginal support staff: 1 Managerial or executive staff: 2 Administrative staff: 1 Paediatricians: 1
5	Perth, Western Australia	15 HCPs	General practitioners: 8 Nurses: 5 AHWs/AHPs: 2
6	Victory Daly, Northern Territory	7 HCPs	General practitioners: 1 Nurses: 2 AHWs/AHPs: 2 Clinical students: 2
7	East Arnhem, Northern Territory	6 HCPs	General practitioners: 1 Nurses: 4 AHWs/AHPs: 1
8	Darling Downs, Queensland	16 HCPs	General practitioners: 5 Nurses: 3 AHWs/AHPs: 3 Managerial or executive staff: 5

Abbreviations: AHP, Aboriginal Health Practitioner; AHW, Aboriginal Health Worker; HCP, health care provider.

### 3.1 Findings

The barriers and facilitators were organised into themes: (i) knowledge, (ii) beliefs and attitudes, (iii) skills and (iv) clinic processes and environment. The classification of themes, subthemes and codes (labels assigned to segments of text that capture their meaning) is illustrated in Figure 2. The codes shown are those found in all or most of the sites. Important codes unique to particular sites are also discussed.

**FIGURE 2:**
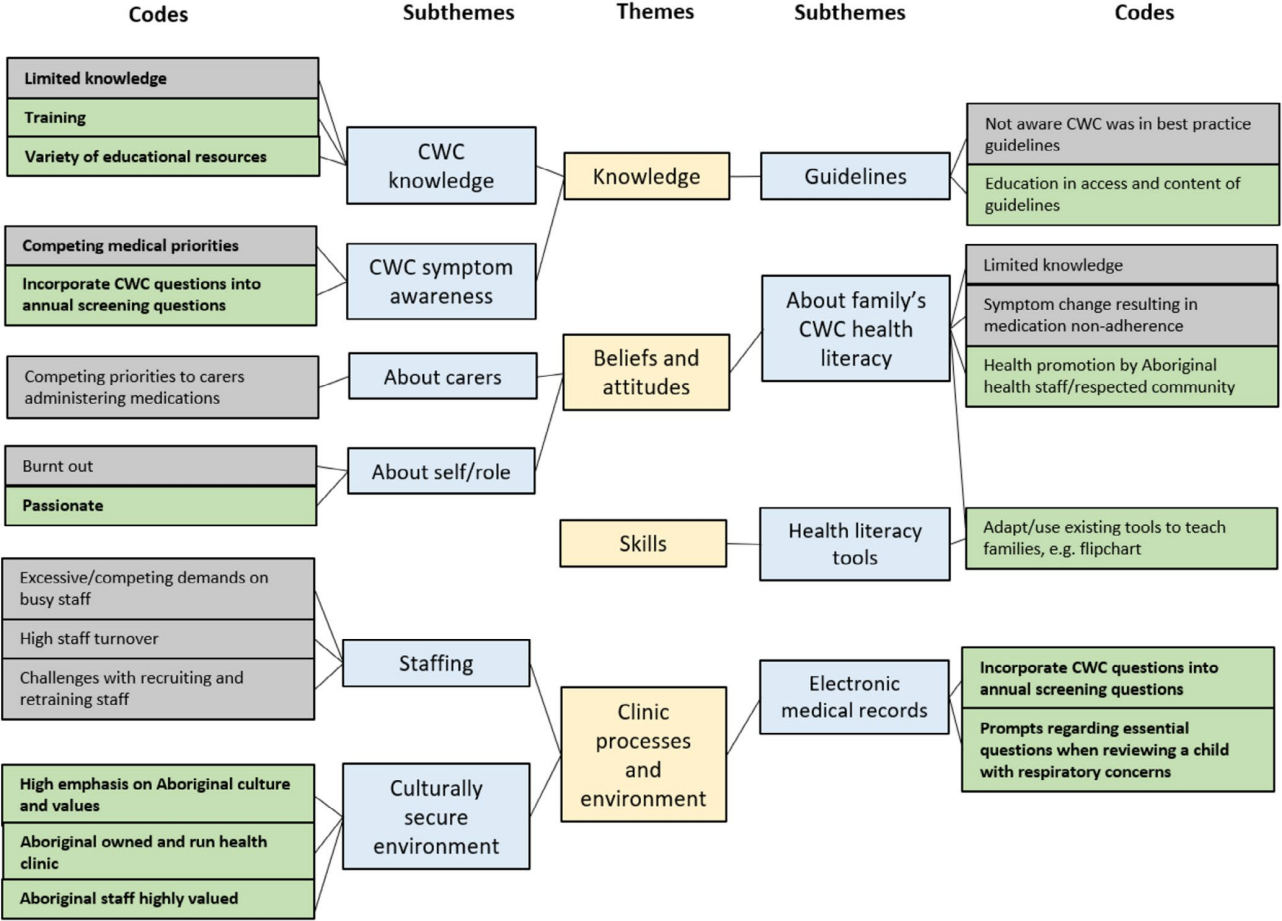
Themes, subthemes and codes identified in most or all sites. CWC, chronic wet cough. Grey = barriers; green = facilitators.

### 3.2 Barriers to Timely Detection and Optimal Management of CWC

#### 3.2.1 Knowledge

##### 3.2.1.1 Limited Knowledge

HCPs in most sites reported having limited knowledge about CWC. However, a minority of HCPs in the East Kimberley sites indicated they were aware of CWC due to previous information sessions conducted by the research team in another town in the region [5].

I personally feel that clinic staff are not across this [CWC] and do not know where to go [for help].—Nurse, Site 1

##### 3.2.1.2 CWC Symptom Awareness

HCPs believed that CWC was likely more widespread than they were identifying, as children often had multiple medical concerns, limiting the time available to manage each issue.

They [the child] often don't come with just wet cough. They come with their anaemia and scabies and other things that take up a lot of the consult.—General practitioner, Site 5

##### 3.2.1.3 Guidelines

Most HCPs reported that their clinic used multiple guidelines, with individual staff members relying on different ones. Although HCPs were familiar with these guidelines, many were unaware they contained information on managing CWC. In some communities, HCPs found it challenging to locate relevant information on CWC because it was categorised under a broad heading and easily overlooked.

There are so many guidelines and protocols that I think people are drowning in them.—General practitioner, Site 6

#### 3.2.2 Beliefs and Attitudes

##### 3.2.2.1 About Carers

HCPs reported varying levels of community awareness and understanding of CWC. They acknowledged the effort required from families to treat PBB that typically requires administering antibiotics twice a day for two or more weeks. They identified many potential challenges and competing priorities for caregivers. HCPs also acknowledged the challenges carers experience when administering therapy to children who may have difficulties tolerating medications.

There is often a lot going on in their [Aboriginal families] lives and then to give them something else to do is just too much, more than they can cope with, that's a hard one.—Nurse, Site 6

Additional challenges were identified in remote sites. HCPs recognised that the first‐line antibiotic used for PBB needs to be refrigerated, but many homes did not have a working fridge. In addition, HCPs noted that families, especially in remote areas, may often move between communities due to extensive cultural connections and may no longer be in the community when follow‐up appointments are needed.

##### 3.2.2.2 About Family's CWC Health Literacy

HCPs reported that their interactions with families indicated limited awareness of CWC management. Specifically, they noted that some families may discontinue antibiotics prematurely when the child shows signs of improvement.

I know that duration is a problem in community, not because they have a resistance to duration, but often they [families] start going ‘oh they feeling better I don't need that anymore’.—Member of the clinic management team, Site 8

##### 3.2.2.3 About Self/Role

Many HCPs reported experiencing burnout, particularly in remote communities, often due to overwhelming workloads and the expectation to take on responsibilities beyond their typical duties.

There is burnout for the health workers and the nurses out in remote. I am amazed at what they [nurses] do here. They do so much more than they should, way beyond, and it burns them out.—Aboriginal Health Worker (AHW), Site 1

#### 3.2.3 Clinic Processes and Environment

##### 3.2.3.1 Staffing

HCPs in nearly all sites reported facing excessive and competing demands on staff. In addition, HCPs described difficulties in recruiting and retaining staff, leading to high turnover and further exacerbating staffing challenges.

The place [clinic] is so busy, you are just so stretched and run off your feet … We are so short‐staffed.—Nurse, Site 1

##### 3.2.3.2 Prescribing Medications

In some very remote communities, the intermittent presence of doctors was identified as a barrier to diagnosing and treating PBB. Since treatment for PBB typically involves prescribing antibiotics, families needed to visit the clinic at specific times when a doctor was present, an arrangement that could be challenging if the family was unavailable or had limited transport.

### 3.3 Participant Proposed Facilitators to Timely Detection and Optimal Management of CWC

#### 3.3.1 Knowledge

##### 3.3.1.1 Training and Educational Resources

The primary facilitator identified by HCPs was training in detection and management of CWC. HCPs emphasised the importance of repeated exposure to information and had a preference for diverse learning formats, including face‐to‐face sessions and online learning that could be accessed at their convenience. All HCPs expressed a strong desire for training opportunities and valued a variety of educational resources such as quick reference flowcharts, podcasts and mobile applications.

We need to inform health professionals … We need to be aware of this [about CWC].—General practitioner, Site 4

##### 3.3.1.2 Guidelines

HCPs explained that guidelines must be useable and accessible. HCPs also expressed a need for education on both content and accessibility of relevant guidelines that could be incorporated into training sessions, ensuring that staff not only understand the guidelines but know where to find them and apply them effectively in clinical practice.

#### 3.3.2 Beliefs and Attitudes

##### 3.3.2.1 About Family's CWC Health Literacy

HCPs emphasised the importance of health promotion in enhancing families' health literacy about CWC. Some clinics already had dedicated health promotion teams and were enthusiastic about incorporating CWC education into their existing initiatives, ensuring that families received clear, accessible and culturally secure information.

Our people, they haven't been educated [about CWC] … They need to be taught about this and what will happen and so you have to show them.—AHW, Site 1

##### 3.3.2.2 About Self/Role

HCPs generally expressed a strong sense of fulfilment in their roles and a deep commitment to serving their communities. Their dedication was particularly evident in the desire to support families in remote areas, ensuring they had access to education and resources needed for better health outcomes. Most HCPs expressed the value they place on collaboration with other HCPs in providing the best care for families.

I am motivated and passionate. I have a passion to work with the younger ones with the families. I want to give them a healthy start.—AHW, Site 1

#### 3.3.3 Clinic Processes and Environment

##### 3.3.3.1 Electronic Medical Records

Clinicians identified system changes to improve CWC detection, such as embedding CWC questions into annual health checks, ensuring that screening for CWC became a standard part of care and facilitate the opportunistic detection of CWC. HCPs suggested electronic medical record prompts to guide assessment of children presenting with respiratory illness.

##### 3.3.3.2 Culturally Secure Environment

HCPs emphasised the importance of Aboriginal culture and values, as well as Aboriginal‐owned and operated health clinics, in fostering a culturally secure environment. A key component was the role of AHWs and Aboriginal Health Practitioners (AHPs), whose value was paramount in building trust and improving communication between patients and healthcare providers.

I think for our practice and most Aboriginal medical services, the AHWs are key to leveraging and being that advocate for the patients and the support person.—Member of the clinic management team, Site 8

##### 3.3.3.3 Medication Adherence

HCPs emphasised the importance of providing health information to families, particularly regarding completing the full course of prescribed antibiotics for suspected PBB. In remote communities, strategies such as clinic‐assisted medication administration through home visits or arranging transport to clinic were raised to help ensure that children receive their medication.

##### 3.3.3.4 Medication Prescription

In the extremely remote communities of Western Australia, given the challenges in having a permanent doctor on site, nurses and AHPs wanted to be empowered with knowledge and skills to initiate treatment for suspected PBB.

## 4 Discussion

In this study exploring barriers and facilitators to the detection and optimal management of CWC from the perspectives of HCPs, interviews with 83 practitioners across eight sites identified several key findings. HCPs identified limited condition‐specific knowledge, time and competing priorities as the most salient barriers to timely identification and optimal management of CWC in Aboriginal and Torres Strait Islander children. HCPs expressed the need for more training, educational resources and various clinic system changes to ease the process in the time‐constrained environment in which they work—improvements to electronic medical record functions, incorporating CWC into screening questionnaires, improving accessibility of guidelines, improved access to medication prescribing and the clinic assisting families with medication administration. In addition, HCPs highlighted the importance of a culturally secure environment to facilitate engagement with patients and families. Although the barriers and facilitators were broadly similar across all settings, context‐specific tailoring of solutions would be required to overcome barriers in different settings.

Most HCPs reported having limited knowledge of CWC even though the symptom is highly prevalent in Aboriginal and Torres Strait Islander children [22] and could signal an underlying or serious progressive disease [23]. Our findings align with our previous, single‐centre study exploring clinician perspectives [3] and other studies indicating that HCPs often do not recognise the significance of CWC [4, 24]. One possible reason for this limited awareness is that PBB, a common cause of CWC [25, 26], was only clearly described in the medical literature nationally in 2006 [27] and internationally in 2008 [28]. Training in undergraduate courses and continued medical education is needed to increase clinician knowledge and awareness. Such training must raise awareness of national clinical practice guidelines for managing CWC [29] and culturally secure healthcare provision in respiratory medicine. To this end, e‐learning modules hosted by peak bodies provide accessible, flexible and cost‐effective options [30].

Time pressures in the workplace were a major barrier, and HCPs expressed concern about burnout (particularly those in remote sites), demonstrating the interplay between multiple barriers. Clinician burnout, driven by high demands on a constrained workforce, limited resources and the pressure to manage diverse health issues, negatively impacts staff well‐being and service delivery [31]. HCPs suggested multiple system changes that would increase efficiency and ease workload while enabling them to provide better care. Electronic medical records with built‐in prompts can reduce cognitive burden on HCPs [32] who must manage a vast array of medical conditions. Adding questions about CWC into the government‐funded annual review (Medicare Benefits Schedule Item 715) health assessment for Aboriginal and Torres Strait Islander people allows for opportunistic detection [1]. In remote communities with limited access to doctors, initiatives that safely empower AHWs/AHPs to initiate appropriate treatment for suspected PBB help combat the higher morbidity and mortality associated with living in rural and remote areas where doctors are not often physically present [33].

HCPs in this study emphasised the importance of culturally secure care and the vital role of AHWs/AHPs, consistent with existing literature [34, 35, 36]. AHWs/AHPs provide essential cultural knowledge and strong community connections, improving the quality of health care for families [36]. These perspectives highlight the need for systemic change and a coordinated, structured approach to care, where Aboriginal patients feel respected and understood (i.e., achieving cultural security) [37].

Our study is important as it represents a comprehensive assessment of barriers and facilitators from the perspective of clinicians and clinic staff across a broad spectrum of Australian settings, ranging from small, highly remote communities to major metropolitan cities. Complementing the viewpoints of carers, clinician insights are crucial for delivering a comprehensive healthcare service.

### 4.1 Limitations

One limitation of our study is that it did not represent all Australian states, territories or Aboriginal and Torres Strait Islander communities. In addition, the majority of the primary care clinics (seven out of eight) were ACCHOs, with only one being government‐controlled, which may affect the generalisability of our findings. However, our study included eight diverse sites across three states and territories, and key barriers and facilitators identified were consistent across sites, suggesting broader applicability. Therefore, translation to government‐run clinics or other regions would likely be successful with consideration of contextual differences in service delivery, governance and patient populations. Another potential limitation is that the perspectives captured may not fully represent all viewpoints, as participation was voluntary. However, nearly all HCPs in each site took part. Some HCPs may have felt hesitant to discuss specific topics in a group setting or in the presence of their line manager. Despite this, insights gained from individual interviews closely aligned with those shared in group discussions, supporting the reliability of the findings.

### 4.2 Conclusion

Limited clinician knowledge on CWC, time constraints and competing priorities are important barriers to the timely detection and management of CWC in Aboriginal children. Although the barriers and facilitators identified were largely consistent across settings, potential solutions require context‐specific tailoring, with differences primarily related to community size, availability of existing infrastructure and systems and local preferences. Comprehensive efforts are needed to improve clinicians' management of CWC and to ensure health care for Aboriginal and Torres Strait Islander people is provided with cultural security. National health policies should centre culturally secure, context‐specific and community co‐designed approaches to care delivery and governments should consider resourcing workforce development and system‐level supports to enable effective implementation of health initiatives for Aboriginal people.

## Author Contributions

Conceptualisation: A.S., R.W., A.B.C. Data curation: G.T.Y.L., A.F., A.S. Formal analysis: G.T.Y.L., M.K., M.T., P.L., R.A., E.R.B., N.J., G.B.M., A.S. Funding acquisition: A.S., A.B.C., R.A., G.B.M., P.M., R.N., M.T., R.W., A.B.C. Investigation: G.T.Y.L., R.A., L.B., P.L., E.R.B., M.K., G.B.M., S.S., M.T., J.E., R.K., J.W., C.D., Aa.S., A.S. Methodology: A.S., A.B.C., R.A., G.B.M., P.S.M., R.N., M.T., R.W., A.B.C. Project administration: G.T.Y.L., A.F., A.S. Supervision: A.S., R.A., G.B.M., A.B.C., R.W. Writing (original draft): G.T.Y.L. Writing (review and editing): All authors.

## Funding

An NHMRC Partnership Grant (1170735) funded the research expenditure costs and salaries of program managers and local researchers. A.S. received salary support from Medical Research Future Fund Investigator Grant (1193796). A.B.C. received salary support from National Health and Research Council Investigator Grant (2025379).The funding sources had no role in planning, writing or publication of the work, or for a research paper, any role in study design, data collection, analysis or interpretation, reporting or publication.

## Disclosure

Not commissioned; externally peer reviewed.

## Conflicts of Interest

The authors declare no conflicts of interest.

## Supporting information


**Table S1:** Interview guide.
**Table S2:** Interviewer demographics and interview periods for each site.
**Table S3:** COREQ (COnsolidated criteria for REporting Qualitative research) Checklist.

## Data Availability

The participants of this study did not give written consent for their data to be shared publicly, so due to the sensitive nature of the research, supporting data are not available.
